# Conserved in-ovo cranial ossification sequences of extant saurians allow estimation of embryonic dinosaur developmental stages

**DOI:** 10.1038/s41598-020-60292-z

**Published:** 2020-04-09

**Authors:** Kimberley E. J. Chapelle, Vincent Fernandez, Jonah N. Choiniere

**Affiliations:** 1 Evolutionary Studies Institute, University of the Witwatersrand, Johannesburg, Gauteng, South Africa; 2 School of Geosciences, University of the Witwatersrand, Johannesburg, Gauteng, South Africa; 3 European Synchrotron Radiation Facility, Grenoble, France; 4 Imaging and Analysis Centre, Natural History Museum, London, United Kingdom

**Keywords:** Evolutionary developmental biology, Palaeontology

## Abstract

Dinosaur embryos are among the rarest of fossils, yet they provide a unique window into the palaeobiology of these animals. Estimating the developmental stage of dinosaur embryos is hindered by the lack of a quantitative method for age determination, by the scarcity of material, and by the difficulty in visualizing that material. Here we present the results of a broad inquiry, using 3D reconstructions from X-ray computed tomography data, into cranial ossification sequences in extant saurian taxa and in well-preserved embryos of the early branching sauropodomorph dinosaur *Massospondylus carinatus*. Our findings support deep-time conservation of cranial ossification sequences in saurians including dinosaurs, allowing us to develop a new method for estimating the relative developmental percentage of embryos from that clade. We also observe null-generation teeth in the *Massospondylus carinatus* embryos which get resorbed or shed before hatching, similar to those of geckos. These lines of evidence allow us to confidently estimate that the *Massospondylus carinatus* embryos are only approximately 60% through their incubation period, much younger than previously hypothesized. The overall consistency of our results with those of living saurians indicates that they can be generalized to other extinct members of that lineage, and therefore our method provides an independent means of assessing the developmental stage of extinct, in-ovo saurians.

## Introduction

Birds are the sole living dinosaur lineage and are therefore often used as a modern analogue when studying dinosaurian evolution, especially where fossilized remains are lacking, such as in developing eggs^1–6^. Embryology has revealed that some non-avian dinosaurs display both plesiomorphic developmental characteristics (such as a slow incubation period) as well as derived developmental characteristics of birds (such as skeletal anatomy and egg macro- and microstructure)^7^. Recent research has also shown that the shape of the bird skull can be explained by paedomorphic retention of juvenile, non-avian dinosaur features^4^. Despite these findings, little is known about the cranial ossification sequence of dinosaurian embryos, and how this sequence compares to other saurians^8,9^.

Ossification patterns during embryonic development have been studied using clearing and staining for a variety of saurian taxa including: several species of galliform^10,11^, palaeognath^12^, anseriform^11^, and passeriform birds^11^; crocodilians^13^; testudines^14–18^; and squamates^19–21^. More recently, X-ray micro-computed tomography (µCT) has also been used as a method to look at these patterns^22,23^. In birds, the general ossification sequence of skeletal elements in embryos has been found to be conserved within species and to a certain degree between species and groups, regardless of altriciality or precociality^24,25^ in hatchlings. However, some heterochronies can occur with the relative timing of these ossification events varying slightly^11,12^. In non-avian reptiles, there is very slight interspecific and intraspecific variability in both the pattern and timing of cranial ossification^13,14,18–21,26^. Understanding these ossification sequences as well as the phylogenetic relationships between the taxa is important for the clarification of heterochronic processes in macroevolution.

In extant taxa, embryonic stages are commonly determined by the appearance of morphological traits such as somites, cartilaginous processes, pigmentation, muscles, brain development, etc.^15,27–30^. The level of cranial ossification has been previously mentioned for some of these stages (for example in crocodiles and some squamates), but the ossification sequence of the bones has not been explicitly used nor universally applied as a criterion for assessing developmental stage^23,29,30^. However, a broader study using event-pair cracking^31^, found that there are five modules in the cranial ossification sequence across large phylogenetic distances^32^. These consist of the jaw bones, the palatal bones, the bones forming the orbit, the skull roof bones and the braincase bones.

In extinct saurians, dinosaurs have the most abundant embryonic record, with in-ovo fossils having been found across all three major lineages^33–41^. Most dinosaur embryonic material includes cranial bones. Past studies have used the degree of cranial sutural closure to estimate the level of maturity of these dinosaur embryos^42–44^. The underlying assumption of these studies is that, as animals mature, the sutures become narrower and eventually close^8,9^. This method is somewhat compromised by post-mortem disarticulation and slow growth rates of some reptiles^9^. The progressive closure of sutures is also not observed in all extant taxa. For example, *Alligator mississippiensis* has cranial sutures that widen throughout ontogeny, possibly due to feeding mechanics^8^. Finally, the degree of sutural closure is difficult to assess in very immature specimens such as embryos.

Other methods of assessing developmental stages in fossil embryos have also been explored. The size of the embryo in proportion to its egg was used to infer maturity in an enantiornithine and in *Massospondylus carinatus*^33,34,41^. A study of therizinosauroid embryos compared the postcranial patterns of ossification to that of alligators^38^. Several studies have compared dinosaur embryonic postcranial and cranial ossification levels to extant birds in order to determine the developmental stage (eg. titanosaurs, oviraptorid, troodontids)^35,36,39^. Incubation periods are a complicating factor for such studies, as they vary greatly between living saurians (i.e., birds, crocodilians, and turtles)^7^, making it challenging to determine which clade is the best proxy for dinosaurs. To our knowledge, there is no study that compares ossification levels of individual cranial bones in dinosaurs to a broad sample of saurian embryos.

In 1976, a clutch of seven subspherical eggs (BP/1/5347a) was discovered by Prof. James Kitching in the early Jurassic upper Elliot Formation of Golden Gate Highlands National Park, South Africa^34,45–47^. Two partially exposed embryos in the clutch were quickly identified as being dinosaurian^47^, making them among the oldest known dinosaur eggs and embryos in the world. These eggs were later identified as belonging to the basal sauropodomorph dinosaur species *Massospondylus carinatus*^34^ and the visible embryonic remains were described. The size of the embryos relative to their respective eggs, along with general observations about the level of ossification, and the presence of a stapes and a fourth trochanter lead to the hypothesis that they were nearing the end of development and close to hatching^46^.

Here, we take a new look at embryonic cranial ossification patterns in the *Massospondylus carinatus* embryos (BP/1/5347a). We reconstruct the ossified portions of their embryonic skulls, and we compare them to ossified cranial bones in a growth series of four living saurian taxa (*Gallus gallus*, *Crocodylus niloticus*, *Centrochelys sulcata* and *Pogona vitticeps*) using synchrotron radiation X-ray micro-computed tomography (SRµCT) imaging methods^48^ and published literature^23^. We develop a numerical method for coding the ossification stage of each bone in each specimen, and use a dissimilarity matrix to assess the relative developmental percentage of the *Massospondylus carinatus* embryos.

## Methods

**Figure 1:**
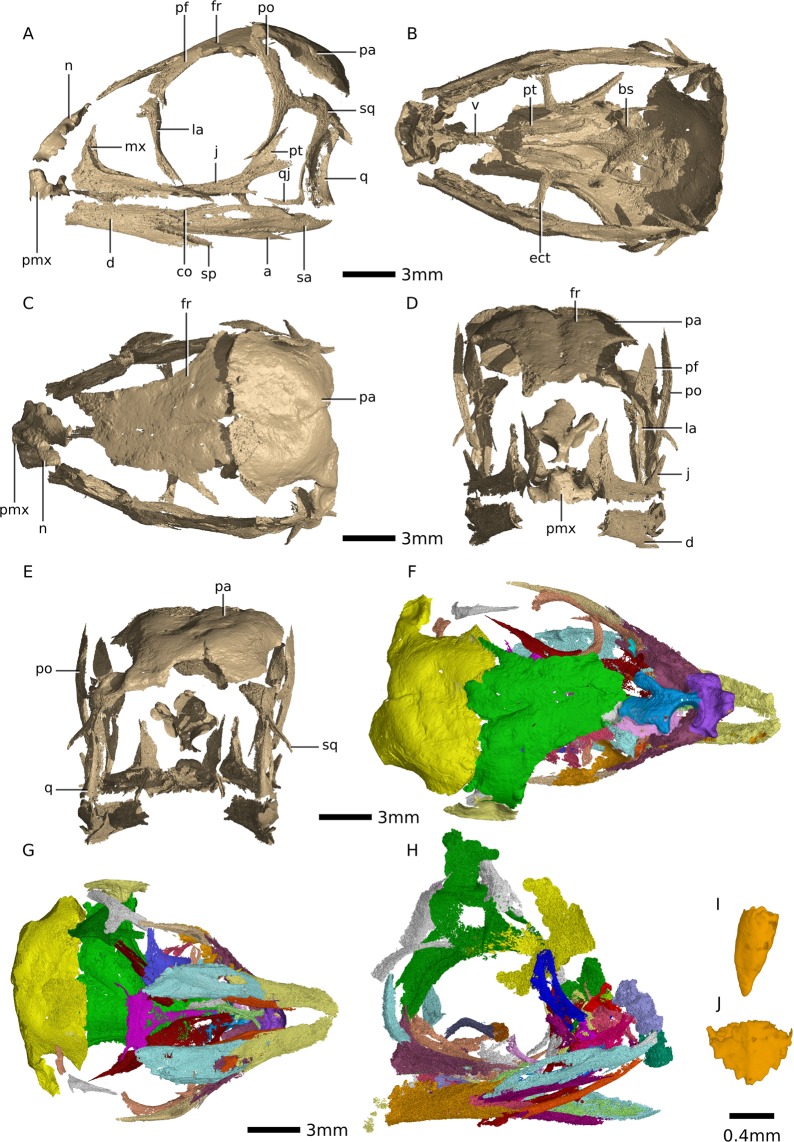
Digital reconstructions of *Massospondylus carinatus* embryonic skulls based on SRµCT data. (**A**) composite of *Massospondylus carinatus* embryonic skulls in left lateral view. (**B**) composite of *Massospondylus carinatus* embryonic skulls in ventral view. (**C**) composite of *Massospondylus carinatus* embryonic skulls in dorsal view. (**D**) composite of *Massospondylus carinatus* embryonic skulls in anterior view. (**E**) composite of *Massospondylus carinatus* embryonic skulls in posterior view. (**F**) *Massospondylus carinatus* Embryo 2 skull reconstruction as preserved in dorsal view. (**G**) *Massospondylus carinatus* Embryo 2 skull reconstruction as preserved in ventral view. (**H**) *Massospondylus carinatus* Embryo 1 skull reconstruction as preserved in lateral view. (**I**) *Massospondylus carinatus* embryonic null-generation tooth. (**J**) *Massospondylus carinatus* functional embryonic tooth. Abbreviations: a, angular; bs, basisphenoid; d, dentary; ect, ectopterygoid; fr, frontal; j, jugal; la, lacrimal; mx, maxilla; n, nasal; pa, parietal; pf, prefrontal; po, postorbital; pmx, premaxilla; pt, pterygoid; sa, surangular; sp, splenial; sq, squamosal; q, quadrate; qj, quadratojugal; v, vomer.

The seven eggs preserved in the clutch BP/1/5347A were characterised at the ID19 beamline of the European Synchrotron Radiation Facility (ESRF, Grenoble, France) using propagation phase contrast SRµCT. Each egg was first imaged individually using a setup providing an isotropic voxel size of 13.11µm. In a second experiment, we focused on the two visible embryonic skulls and increased the resolution using a setup providing an isotropic voxel size of 2.98µm (details of both setups are provided in S1). The bones were digitally reconstructed in VG Studio MAX 3.2 (Volume Graphics, Heidelberg, Germany). The best exemplars of each bone in the *Massospondylus carinatus* embryos were then extracted as surface mesh (.stl) files and combined in order to reconstruct an articulated skull for visualization purposes (Fig. 1). Surface files are available on the online repository Morphosource (https://www.morphosource.org/MyProjects/Dashboard/dashboard/select_project_id/798).

Tomographical data of *Centrochelys sulcata*, *Gallus gallus* and *Crocodylus niloticus* embryos were obtained from the ESRF database (http://paleo.esrf.eu; acquisition parameters provided in Table S2). Each sampled developmental percentage in that dataset (i.e., approximately x% through the incubation period, depending on taxon and specimen availability) was reconstructed for each taxon. Data for *Pogona vitticeps* was obtained from literature^23^ that used µCT scans. It is important to note that this taxon was analysed using the illustrations provided to test our method on published data, that it does not include all developmental percentages in the incubation period, and therefore that it needs to be considered with caution.

The degree of ossification of individual bones was used as an indicator of developmental percentage. We quantified our observations of ossification level across taxa and ontogenetic stages using the following numerical system: stage-code 0 = absence of ossification in that bone; stage-code 1 = beginning of ossification, usually in the form of a small amorphous pellet; stage-code 2 = bone is immediately recognizable in shape, but many of its processes and rami are incompletely ossified, often ending in somewhat ragged projections; and stage-code 3 = bone shape strongly resembles the juvenile condition short of complete expansion (see Table S3).

Bones that are “lost” in some taxa, either through fusion to other bones or lack of complete ossification, were coded as NA in our matrix, although these may have embryonic ossification centres (e.g., the postorbital and prefrontal of *Gallus gallus*^49^). It was not possible to fully track the level of ossification of these (i.e., as stage-code 2 or 3) across the full embryonic sequence.

In two of the *Massospondylus carinatus* embryos, the parietals, frontals and nasals are extremely thin sheets of bone preserved on the surface of the specimen (i.e., they have been freed from the encasing matrix by preparation). The phase retrieval algorithm used for the tomographic reconstruction often obscures the margins of bones located at the surface, making it difficult to digitally reconstruct them (see Fig. 1). They can, however, clearly be seen on the specimen and we have coded them in our ossification level scheme with the aid of visual inspections under a microscope (in this case, as a level 2). As an additional sensitivity analysis, we also coded them as a level 3 and present the results in the supplement (see Table S4).

A matrix was created with each row representing a different relative developmental percentage (days in incubation) of each taxon during incubation (i.e., between the start and end of embryonic ossification, see Table S3) and each column representing a different cranial bone. A dissimilarity distance matrix containing all pairwise comparisons of developmental percentages across all comparable bones using the “Gower” method was computed using R Studio Max v1.1.453^50^ and the package vegan^51^. The “Gower” method was selected as it corrects for missing data (in this case bones which are not present across all taxa) as NAs are not considered when calculating distances^52^. The matrix was used to quantitatively compare dissimilarity between individual stages in extant saurian ontogenetic sequences and the ossification pattern observed in the *Massospondylus carinatus* embryos (see Tables 1 and S5 for distance matrix results using S3, and see S6 for sensitivity analysis distance matrix results using S4). The distance matrix (Table S5) was then subjected to a principal coordinate analysis (PCoA) using the R package ape^53^ (see Table S7 and Fig. S8).

**Table 1:** Dissimilarity distance matrix pairwise comparisons of *Massospondylus carinatus* embryo terminals using the “Gower” method. Specimens in bold represent the shortest distance between Massospondylus carinatus embryos and the extant taxon in question.

	*Massospondylus carinatus*		*Massospondylus cariatus*
*Crocodylus niloticus* 18 days	0,679	*Centrochelys sulcata* 40 days	0,361
*Crocodylus niloticus* 33 days	0,595	*Centrochelys sulcata* 45 days	0,333
*Crocodylus niloticus* 39 days	0,274	*Centrochelys sulcata* 50 days	0,222
*Crocodylus niloticus* 41 days	0,202	*Centrochelys sulcata* 55 days	0,153
*Crocodylus niloticus* 45 days	0,214	***Centrochelys sulcata 61 days***	**0,125**
***Crocodylus niloticus 48 days***	**0,131**	*Centrochelys sulcata* 70 days	0,194
*Crocodylus niloticus* 55 days	0,214	*Centrochelys sulcata* 75 days	0,222
*Crocodylus niloticus* 63 days	0,262	*Centrochelys sulcata* 80 days	0,306
*Crocodylus niloticus* 67 days	0,321		
*Gallus gallus* 11 days	0,240	*Pogona vitticeps* 15 days	0,705
*Gallus gallus* 12 days	0,173	*Pogona vitticeps* 18 days	0,679
*Gallus gallus* 13 days	0,187	*Pogona vitticeps* 24 days	0,538
***Gallus gallus 14 days***	**0,160**	*Pogona vitticeps* 28 days	0,346
*Gallus gallus* 15 days	0,227	*Pogona vitticeps* 32 days	0,282
*Gallus gallus* 16 days	0,240	*Pogona vitticeps* 36 days	0,269
*Gallus gallus* 17 days	0,253	***Pogona vitticeps 48 days***	**0,205**
*Gallus gallus*18 days	0,267	*Pogona vitticeps* 60 days	0,269
*Gallus gallus* 19 days	0,307		

As an additional sensitivity analysis, the stage-code matrix (Table S5) was replaced by a simple presence|absence matrix (binary 0 and 1 stage-codes, see Table S9), and a dissimilarity matrix using the same parameters as above was generated (see Table S10).

## Results

Phase contrast was successful in detecting embryonic remains in BP/1/5347a, as in other studies of fossilized embryonic remains^48^. Our analyses of the clutch using this method revealed skeletal material in only three of the eggs: in the fully prepared embryo in lateral view (here considered to be the bottom left egg), in the prepared embryo in dorsal view, and in the broken egg in the top right corner of the clutch (which included a few cranial bones). Throughout the manuscript, “Embryo 1” will refer to the embryo prepared in lateral view, “Embryo 2” will refer to the embryo prepared in dorsal view and “Embryo 3” will refer to the partial embryo in the top right egg (see Fig. S11).

Embryo 1 is mostly articulated but is missing the anterior tip of its snout. Among its cranial bones, it preserves the maxilla, nasal, jugal, lacrimal, frontal, parietal, postorbital, quadrate, quadratojugal, pterygoid, ectopterygoid, palatine, vomer, basisphenoid, angular, surangular, prearticular, splenial, coronoid and dentary (see Fig. 1 and Table S12).

Embryo 2 is mostly articulated and among its cranial bones preserves the premaxilla (that appears to have been damaged during preparation), maxilla, nasal, jugal, lacrimal, frontal, parietal, postorbital, quadratojugal, pterygoid, ectopterygoid, palatine, vomer, basisphenoid, angular, surangular, prearticular, splenial, coronoid and dentary (see Fig. 1 and Table S12).

Embryo 3 is disarticulated and among its cranial bones only preserves a right maxilla, right postorbital and frontal (see Fig. S13 and Table S12).

In general, the bones that are approximately fully ossified (stage-code 3) in all of the embryos are the bones of the snout (maxilla, premaxilla), the mandible (dentary, coronoid, splenial, surangular, angular, prearticular), the postorbital, prefrontal, quadratojugal, squamosal, as well as some of the bones of the palate (pterygoid, ectopterygoid). The bones of the skull roof (frontal, parietal, nasals) are recognizable in shape but have incompletely ossified margins (stage-code 2), as are the palatine, the vomers, jugals, quadrates, and the lacrimals, in which the full shapes have not been realized.

The only ossified braincase bone in our sample is the basisphenoid (stage-code 2). In Embryo 1, it is a flat sheet of bone with an anteriorly extending cultriform process and a tube-like, partially ossified right basipterygoid process. The left basipterygoid process as well as the basal tubera have not yet ossified. The basisphenoid of Embryo 2 is a flat sheet of bone with a cultriform process but no ossification of the basal tubera or basipterygoid processes. The quadrate is partially ossified in Embryo 1 (portions of the midshaft and proximal portions of the pterygoid and quadratojugal rami have ossified) but is absent in Embryo 2.

All three embryos have teeth in the maxillae, dentaries and premaxilla (where preserved). The teeth have two distinct morphologies: small, simple and conical; and large, broad, and serrated (see Fig. 1). The latter morphology only has crowns or partial crowns formed with no roots. These two morphologies do not appear in any identifiable pattern and can sometimes be found in the same alveolus or in adjacent alveoli (see Fig. S14).

*Crocodylus niloticus*, *Gallus gallus domesticus*, *Centrochelys sulcata* and *Pogona vitticeps* have similar ossification sequences to each other (Tables S3 and S5). The bones of the snout are first to ossify, including the premaxilla, maxilla and dentary. They are followed by the remaining bones of the mandible, excluding the articular, and most of the lateral bones of the face (except for the quadrate and parietal) and the palatal bones (pterygoid, ectopterygoid when present, palatine and vomers). The coronoid (when present) ossifies next, along with the supraoccipital, otoccipital, quadrate, and the basisphenoid. This is also when teeth begin to form (when present). Following this, the basioccipital and prootic begin to ossify, followed by the parietal and laterosphenoid (when present). Finally, the articular and the palpebral (when present) are the last bones to begin ossifying at approximately 70% through the incubation period.

**Figure 2:**
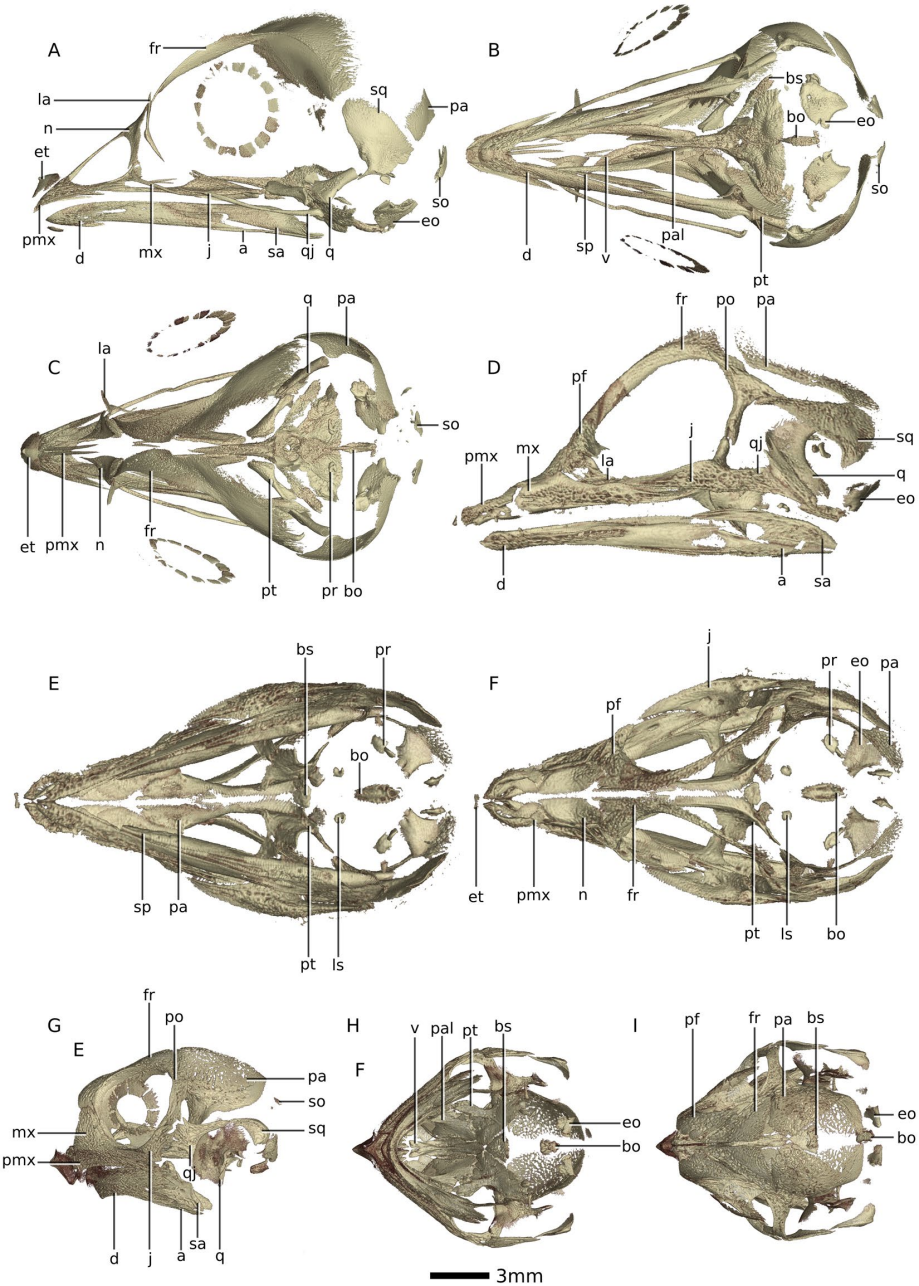
Digital reconstructions of saurian embryo skulls based on SRµCT data. (**A**) *Gallus gallus* at 14 days in incubation period in left lateral view. (**B**) *Gallus gallus* at 14 days in incubation period in ventral view. (**C**) *Gallus gallus* at 14 days in incubation period in dorsal view. (**D**) *Crocodylus niloticus* at 48 days in incubation period in left lateral view. (**E**) *Crocodylus niloticus* at 48 days in incubation period in ventral view. (**F**) *Crocodylus niloticus* at 48 days in incubation period in dorsal view. (**G**) *Centrochelys sulcata* at 61 days in incubation period in left lateral view. (**H**) *Centrochelys sulcata* at 61 days in incubation period in ventral lateral view. (**I**) *Centrochelys sulcata* at 61 days in incubation period in dorsal lateral view. Abbreviations: a, angular; bo, basioccipital; bs, basisphenoid; d, dentary; eo, exoccipital; et, egg tooth; fr, frontal; j, jugal; la, lacrimal; ls, laterosphenoid; mx, maxilla; n, nasal; pa, parietal; pal, palatine; pf, prefrontal; po, postorbital; pmx, premaxilla; pr, prootic; pt, pterygoid; sa, surangular; so, supraoccipital; sp, splenial; sq, squamosal; q, quadrate; qj, quadratojugal; v, vomer.

The dissimilarity matrix (see Tables 1 and S5) indicates that the *Massospondylus carinatus* embryos are most similar to the *Crocodylus niloticus* embryos at 48 days, the *Gallus gallus* embryos at 14 days, the *Centrochelys sulcata* embryos at 61 days of the incubation, and the *Pogona vitticeps* embryos at 48 days of the incubation (see Fig. 2). The distance between the *Massospondylus carinatus* embryos and *Pogona vitticeps* at 48 days is relatively large compared to the others, however this comparison is of lower resolution due to the gap in developmental percentages included in the analysis (no *Pogona vitticeps* embryos between 55% and 74% through the incubation period were illustrated in the published study).

Changing the stage-codes of the *Massospondylus carinatus* embryo frontals and parietals to stage-code 3 does not alter the general results of the dissimilarity matrix (see Table S6). The distance values change, but the smallest distances between *Massospondylus carinatus* and the respective extant taxa remain at the same developmental percentages. The sensitivity analysis using a simple binary presence|absence matrix cannot estimate a developmental percentage for the *Massospondylus carinatus* embryos because too many of the distances between developmental percentages are equal (see Table S10).

## Discussion

Our results show that both the sequence and to a certain extent, the relative timing of cranial ossification are conserved between *Gallus gallus*, *Crocodylus niloticus*, *Centrochelys sulcata* and *Pogona vitticeps*. This sequence agrees with previous work using different methods done on larger phylogenetic samples (including mammals)^31,32,54^. In general, this order follows the aforementioned modules described in the event-pair cracking study^32^ (i.e. jaw bones, the palatal bones, the bones forming the orbit, the skull roof bones and the braincase bones), however some bones ossify later than the rest of their respective modules (such as the coronoid, the laterosphenoid, the articular and the palpebral).

Based on the level of ossification of the basisphenoid, frontals, parietals, palate, and quadrate, as well as the absence of the remaining braincase bones and the articular, we hypothesize that the *Massospondylus carinatus* embryos are approximately 60% through their incubation period (56% or 48 days out of 85 for crocodiles^55^; 67% or 12 days out of 21 for chickens; 61% or 61 days out of 100 for spurred tortoises and 74% or 48 days out of 65 for bearded dragons^56^) (see Fig. 2). In our analysis, the bearded dragon was not scored between 36 days and 48 days in the incubation period (or 55–74%), as this gap was not illustrated in the literature. This comparison therefore has lower precision than the others and is considered with caution. Although at this relative age we would expect the *Massospondylus carinatus* embryos to have an onset of ossification in the rest of the braincase bones (i.e notably exoccipital and basioccipital but also possibly the supraoccipital, prootic and laterosphenoid), the extant taxa present a small ossification centre for these bones at this developmental percentage. It is therefore possible that these had not started to ossify yet in the dinosaur embryos, that the small ossification centres did not preserve, or that they were below the resolution threshold in our phase -contrast -based SRµCT data. This indicates that the embryos are certainly not much more developed than 60% through their incubation period, as these braincase bones would otherwise be more ossified and visible. In *Gallus gallus*, *Crocodylus niloticus,* *Centrochelys sulcata*, and Pogona vitticeps, these braincase bones are all at stage-code 2 at approximately 70–75% through the incubation period (day 15, day 55, day 70 and day 60 respectively, see Table S3). This puts an upper limit on the developmental percentage of the *Massospondylus carinatus* embryos because stage-code 2 ossification is readily seen in our scan in other bones of the skull.

Our hypothesized relative developmental percentage of the *Massospondylus carinatus* embryos indicates that they are earlier in development than previously thought^46^. This makes them some of the ontogenetically youngest dinosaur embryos known. All other dinosaur embryos in the literature with ontogenetic age estimates are hypothesized as being in the last third of their development or near hatching^35–39^. However, analysing the latter using micro computed tomography scans and our stage-code method could reveal that some of them are younger than the *Massospondylus carinatus* embryos presented here.

Our hypothesis for the developmental percentage of the *Massospondylus carinatus* embryos is corroborated by the presence of both null-generation teeth and crowns with adult tooth morphology (see Fig. 1). Although many dinosaur embryos have been found to have teeth^36–38,40^, in all cases the reported morphology is most similar to adult teeth. To our knowledge, null-generation teeth have not been reported in dinosaurs, nor have null-generation and adult teeth been reported in a single embryo. *Troodon* teeth have cylindrical roots, linguobuccaly compressed and mesiodistally elongated crowns. These were hypothesized as being early developmental casts of unossified teeth^35^. *Maiasaura* embryos preserve different generations of teeth, however the budding teeth are hypothesized to grow in the form of the larger teeth preserved. These are therefore replacement teeth and are similar in morphology^57^.

Null-generation teeth form during embryonic development in several reptile species^58–60^. These non-functional teeth are small, unicuspid (even if adult tooth morphology is multicuspid), and possess little or no enamel. They are either resorbed into the jaw or shed into the oral cavity^59^. Little information is available on null-generation teeth in living saurian taxa. However, in geckos, the null-generation teeth appear at about 23% through the incubation period. By 66% into the incubation period, null-generation teeth are present in half of the tooth positions, while the other half are formed by the first functional teeth (null-generation teeth are therefore not a prerequisite for the formation of adult tooth morphologies). By the end of the incubation period, the first set of functional teeth have started to be resorbed while the second set of functional teeth start moving orally^58^. All null-generation teeth are replaced by functional teeth during embryonic development^61^. The simple conical tooth morphology seen in the *Massospondylus carinatus* embryos probably represent null-generation teeth for *Massospondylus carinatus*, which will get resorbed or shed into the oral cavity before hatching. The abundance of these null-generation teeth (approximately half of the teeth preserved, see Fig. S14) correlate well with the 60% in incubation period estimated by the bone ossification.

Our stage-code method provides a simple, relatively precise, repeatable estimate of the developmental percentage in other extinct and extant saurian embryos. Such estimates are of broad utility when including embryos in broader ontogenetic studies. For example, the early developmental percentage of the *Massospondylus carinatus* embryos suggests caution against uncritical use of limb measurements as part of allometric studies, e.g., postural determination. Coupling these developmental percentage estimates with incubation period^7^ also provides better insight into the life histories of dinosaurs and could potentially enable more precise future studies of how birds, for example, decreased their incubation periods.

Although Reisz *et al*. (2010) identified embyronic skeletal material in five of the eggs that comprise BP/1/5347a, our results show that only three of the seven eggs contain embryonic material. Several factors have been hypothesized to have an effect on clutch viability in extant taxa including environmental conditions, eggshell structure, predation and microbial contamination^62,63^. However the patterns of embryonic mortality are poorly understood^63^. There are many potential reasons for fossil eggs being empty, including: a high number of infertile eggs; high levels of early mortality before ossification of the skeleton; leakage from broken eggs during fossilisation; or that the clutch represents two or more clutches laid at different times (Deeming, pers. com.). Given our sample size, we cannot assess any of these critically at this time. However, the three preserved embryos do not differ substantially in their ossification, indicating that they are therefore at similar developmental percentages and are probably from the same clutch. The first and second embryos show some slight differences in level of ossification. The Embryo 1 has a partial basipterygoid process that has started to ossify as well as a quadrate. Embryo 2 does not have either of these elements ossified. Despite these, they are highly synchronous in their development and it is not possible to speculate as to the nature of the depositional time in the nest, if these embryos would have asynchronous hatching, or if these differences represent intraspecific variation in the timing or rate of embryonic ossification^11^.

A strong, highly conserved pattern of cranial ossification is seen in saurians. Both this pattern and the level of ossification at the different developmental percentages are enough to correlate and approximate the *Massospondylus carinatus* embryos. However, several complicating factors need to be taken into account with regards to the comparative extant embryonic datasets. Because X-ray µCT imaging of extant embryos can involve sacrificing the specimens, the series we present for these taxa do not track the ossification sequence of one single individual, but rather of several individuals (one for each developmental percentage represented). Previous research has found that there is slight inter-individual variations in the timing of ossification^11^. However, given the strength of the overall pattern, we do not consider the variance introduced by these minor differences to compromise our overarching result. The datasets for *Crocodylus niloticus, Centrochelys sulcata* and *Pogona vitticeps* do not include individuals for every day in the incubation period, but rather individuals which are several days apart (especially in *Pogona vitticeps*). This reduces precision for tracking the ossification sequence, complicates understanding ossification rates in individual bones, and represents a fertile area for further study.

## Conclusion

Cranial ossification sequence is conserved in saurians, including in dinosaurs, even across large phylogenetic distances. Using this observation and a stage-code based method recording the ossification level of each cranial bone at each developmental percentage during the incubation allows for the determination of the developmental percentage of fossil embryos. Based on 3D reconstructions from SRµCT data, we find that the famous embryos of *Massospondylus carinatus* (BP/1/5347a) are approximately 60% through their incubation period, much earlier than previously hypothesized. This is corroborated by the presence of formerly unreported null-generation teeth. This research provides potential to elucidate broader patterns of macroevolution and suggests that heterochronic shifts in ossification timing are likely not a major mechanism for how different shaped skulls evolved.

## Supplementary information

Supplementary Information S1.

Supplementary Table S2.

Supplementary Table S3.

Supplementary Table S4.

Supplementary Table S5.

Supplementary Table S6.

Supplementary Table S7.

Supplementary Figure S8.

Supplementary Table S9.

Supplementary Table S10.

Supplementary Figure S11 to S14.

## Data Availability

Tomographical data of *Centrochelys sulcata*, *Gallus gallus* and *Crocodylus niloticus* embryos are available on the ESRF database (http://paleo.esrf.eu). Surface files of the embryos presented in Fig. 2 have been uploaded to Morphosource (https://www.morphosource.org/MyProjects/Dashboard/dashboard/select_project_id/798). Tomographical data of the *Massospondylus carinatus* embryos (BP/1/5347a) are property of the University of the Witwatersrand. Surface files are available on the online repository Morphosource (https://www.morphosource.org/MyProjects/Dashboard/dashboard/select_project_id/798).
