# Supplementary material for: Conserved in-ovo cranial ossification sequences of extant saurians allow estimation of embryonic dinosaur developmental stages
Source: Sci Rep. 2020 Apr 9;10:4224. doi: 10.1038/s41598-020-60292-z (PMC7145871; doi:10.1038/s41598-020-60292-z)
Supplement: Supplementary file 1 — Supplementary Information S1. [file 41598_2020_60292_MOESM1_ESM.docx]

Conserved in-ovo cranial ossification sequences of extant saurians allow estimation of embryonic dinosaur developmental stages

KIMBERLEY E. J. CHAPELLE^1,2^*, VINCENT FERNANDEZ^1,3,4^, JONAH N. CHOINIERE^1^

^1^ Evolutionary Studies Institute, University of the Witwatersrand, Johannesburg, Gauteng, South Africa

^2^School of Geosciences, University of the Witwatersrand, Johannesburg, Gauteng, South Africa

^3^ European Synchrotron Radiation Facility, Grenoble, France

^4^ Imaging and Analyses Centre, Natural History Museum, London, United Kingdom

[*kimi.chapelle@gmail.com](mailto:*kimi.chapelle@gmail.com)

**Supplementary information**

**S1.** Scanning parameters for *Massospondylus carinatus* embryos.

The seven eggs preserved on the clutch BP/1/5347A were characterised at the ID19 beamline of the European Synchrotron Radiation Facility (ESRF, Grenoble, France) using propagation phase contrast X-ray micro computed tomography. For the first part of the experiment, we imaged whole eggs individually using a setup generating data with a voxel size of 13.11 µm. This would allow for visualising the full skeleton of the two embryos visible from previous preparation and to inspect unprepared eggs for potential presence of bones. In a second part, we changed the setup to increase the resolution, focusing on the skulls of the two prepared embryos in order to get more details from the minute bones. This second setup generated data with a voxel size of 2.98 µm.

The X-ray beam configuration was similar for both setups: white beam from a wiggler W150B filtered with a 1 mm diamond window, 2.8 mm of Aluminium, 12 mm of Copper and 0.3 mm of Tungsten. The wiggler gap was adapted depending on the detector setup: 32 mm gap for the 13.11 µm setup (detected total integrated energy of 175 keV) and 37 mm gap for the 2.98 µm setup (detected total integrated energy of 166 keV).

The 13.11 µm setup consisted of an indirect detector comprising a 500 µmm LuAG scintillator (Lutetium Aluminium Garnet), a 1x magnification from a set of two 100 mm Hasselblad photographic lenses (Hasselblad, Gothenburg, Sweden) and a FReLoN 2K CCD camera (developed at the ESRF). With this setup, the detector was placed 11 m downstream of the sample to maximize phase contrast imaging by free space propagation. The 2.98 µm setup consisted of an indirect detector comprising a 200 µm LuAG scintillator, a set of two Hasselblad photographic lenses (100 mm and 210 mm, 2.1x magnification) and a PCO.edge 5.5 sCMOS with camera link (PCO, Kelheim, Germany). This detector was located 7.5 m downstream from the sample. For both setup, the rotation axis was shifted near one edge of the recorded projection, resulting in almost doubling the reconstructed field of view (ie. Half acquisition protocol, Carlson et al. (2011)).

For the 13.11 µm setup, 4998 projections were recorded over a 360° rotation of the sample (necessary for half acquisition protocol). The exposure time was 0.1 s per projection. For the 2.98 µm setup, 8000 projections were recorded over a 360° rotation of the sample. The higher number of projection was chosen to compensate for the higher noise of the PCO.edge 5.5 camera compared to the FReLoN 2k. Exposure for this setup was 0.25 s per projection.

For both setups, several acquisitions were necessary on the vertical axis to cover the full height of the scanned region. The sample was moved on the vertical axis by a distance ranging from 33% to 50% of the vertical field of view. The important overlap was subsequently used to concatenate the scans, using a weighted average, limiting artefact that could be caused by the beam profile.

For the imaging of the skulls using the 2.98 µm setup, several scans were also necessary on the horizontal axis. The sample was moved on the horizontal axis and final 16 bits volumes were stitched using a weighted average on the overlapping part.

Tomographic reconstruction was performed using PyHST2 (Mirone et al., 2014) using the single distance phase retrieval approach (Paganin et al., 2002). The obtained 32-bits data were converted to 16-bits stack of jpeg2000 (compression of 10), using the 3D histogram from PyHST2 and a saturation of 0.002% of the 32-bits data. Additional processing included ring correction (Lyckegaard et al., 2011) and cropping of the volume to limit the size of the data. For the data produce with the 2.98 µm setup, general grey level distribution was affected by the local tomography condition (inspected region much smaller than the size of the object). This was corrected by normalizing the data against a version of the data on which we applied a low pass filter (obtained with a gaussian blur with a radius of 100-200 pixels depending on the case).

Carlson K.J., Stout D., Jashashvili T., De Ruiter D.J., Tafforeau P., Carlson K.& Berger L.R. **2011**. The endocast of MH1, *Australopithecus sediba*. *Science.* 333, 1402-1407.

Lyckegaard A., Johnson G.& Tafforeau P. **2011**. Correction of ring artifacts in X-ray tomographic images. *Int. J. Tomo. Stat.* 18, 1-9.

Mirone A., Brun E., Gouillart E., Tafforeau P.& Kieffer J. **2014**. The PyHST2 hybrid distributed code for high speed tomographic reconstruction with iterative reconstruction and a priori knowledge capabilities. *Nuclear Instruments and Methods in Physics Research Section B: Beam Interactions with Materials and Atoms.* 324, 41-48.

Paganin D., Mayo S., Gureyev T.E., Miller P.R.& Wilkins S.W. **2002**. Simultaneous phase and amplitude extraction from a single defocused image of a homogeneous object. *Journal of microscopy.* 206, 33-40.
