# Supplementary material for: Conserved in-ovo cranial ossification sequences of extant saurians allow estimation of embryonic dinosaur developmental stages
Source: Sci Rep. 2020 Apr 9;10:4224. doi: 10.1038/s41598-020-60292-z (PMC7145871; doi:10.1038/s41598-020-60292-z)
Supplement: Supplementary file 8 — Supplementary Figure S8. [file 41598_2020_60292_MOESM8_ESM.pdf]

Conserved in-ovo cranial ossification sequences of extant saurians allow estimation of embryonic dinosaur developmental stages

KIMBERLEY E. J. CHAPELLE[1,2]*, VINCENT FERNANDEZ[1,3,4], JONAH N. CHOINIERE[1]
[1] Evolutionary Studies Institute, University of the Witwatersrand, Johannesburg, Gauteng, South Africa
[2]School of Geosciences, University of the Witwatersrand, Johannesburg, Gauteng, South Africa
[3] European Synchrotron Radiation Facility, Grenoble, France
[4] Imaging and Analyses Centre, Natural History Museum, London, United Kingdom
*kimi.chapelle@gmail.com

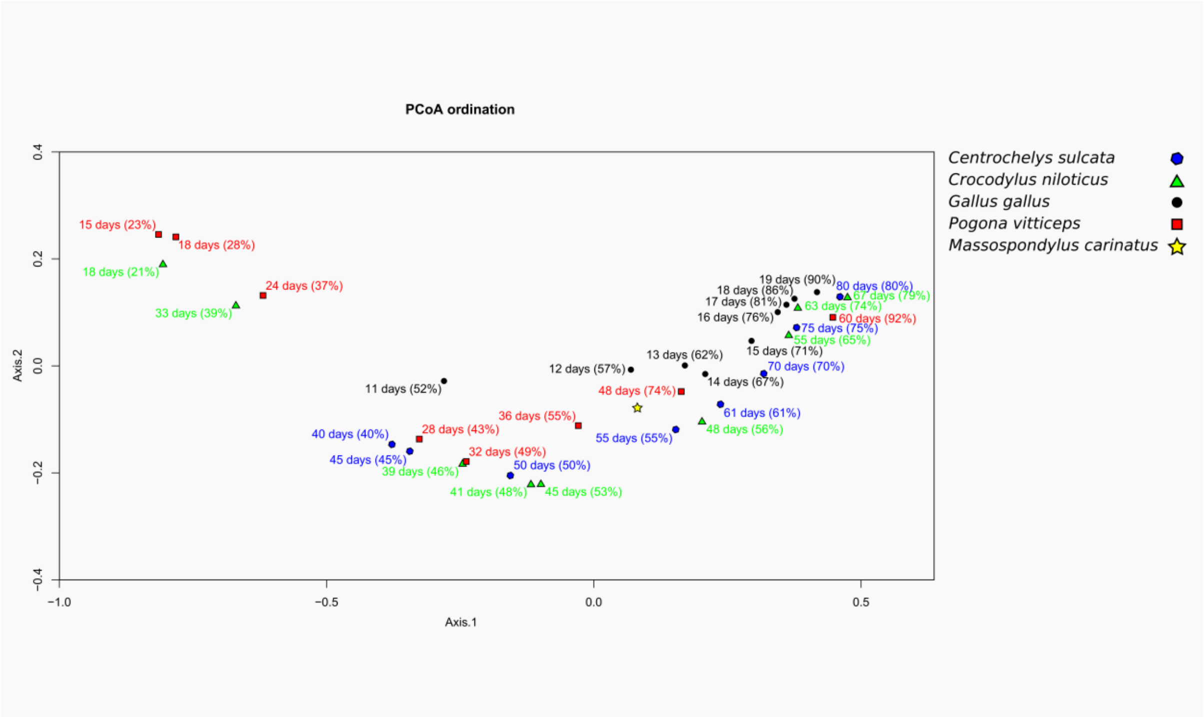

Figure S8. PCoA ordination plot.
