# Supplementary material for: Conserved in-ovo cranial ossification sequences of extant saurians allow estimation of embryonic dinosaur developmental stages
Source: Sci Rep. 2020 Apr 9;10:4224. doi: 10.1038/s41598-020-60292-z (PMC7145871; doi:10.1038/s41598-020-60292-z)
Supplement: Supplementary file 11 — Supplementary Figure S11 to S14. [file 41598_2020_60292_MOESM11_ESM.pdf]

Conserved in-ovo cranial ossification sequences of extant saurians allow estimation of embryonic dinosaur developmental stages

KIMBERLEY E. J. CHAPELLE[1,2]*, VINCENT FERNANDEZ[1,3,4], JONAH N. CHOINIERE[1]
[1] Evolutionary Studies Institute, University of the Witwatersrand, Johannesburg, Gauteng, South Africa
[2]School of Geosciences, University of the Witwatersrand, Johannesburg, Gauteng, South Africa
[3] European Synchrotron Radiation Facility, Grenoble, France
[4] Imaging and Analyses Centre, Natural History Museum, London, United Kingdom
*kimi.chapelle@gmail.com

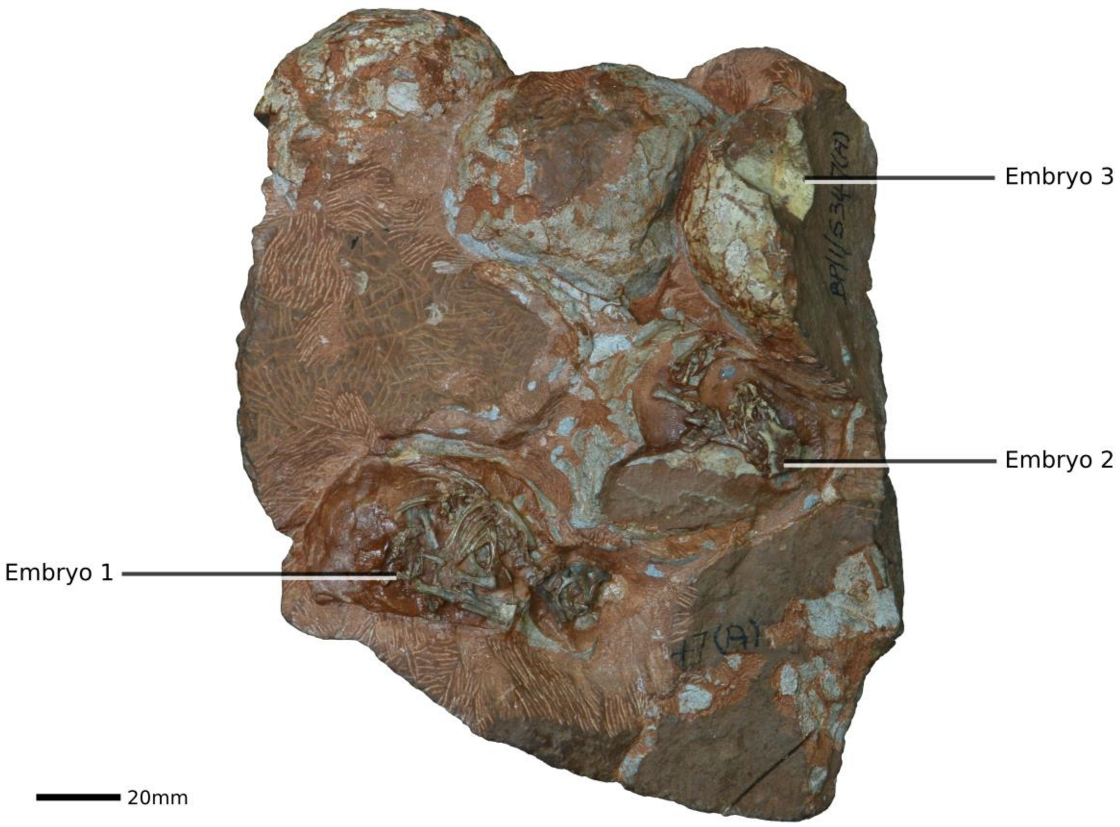

Figure S11. *Massospondylus carinatus* clutch photograph (BP/1/5347a).

Table S12. Bones preserved in each *Massospondylus carinatus* embryo.

Bone	Embryo 1	Embryo 2	Embryo 3
premaxilla	broken	X	
maxilla	X	X	X
dentary	X	X	
coronoid	X	X	
splenial	X	X	
surangular	X	X	
angular	X	X	
articular			
nasal	X	X	
prefrontal	X	X	
postorbital	X	X	X
jugal	X	X	
quadratojugal	X	X	
squamosal	X	X	
frontal	X	X	X
parietal	X	X	
supraoccipital			
otoccipital			
columella			
prootic			
basioccipital			
basisphenoid	X	X	
laterosphenoid			
vomer	X	X	

palatine	X	X	
pterygoid	X	X	
ectopterygoid	X	X	
quadrate	X	X	
teeth	X	X	X

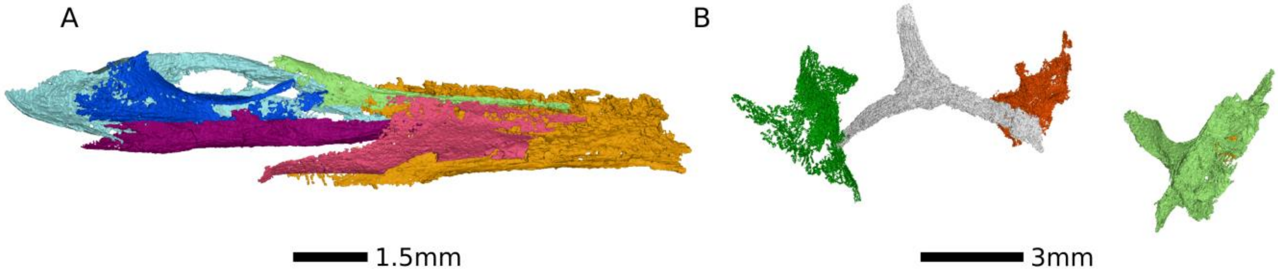

Figure S13. *Massospondylus carinatus* embryo cranial bone reconstructions as preserved (BP/1/5347a). A, Embryo 1 left mandible in medial view; B, Embryo 3 cranial bones.

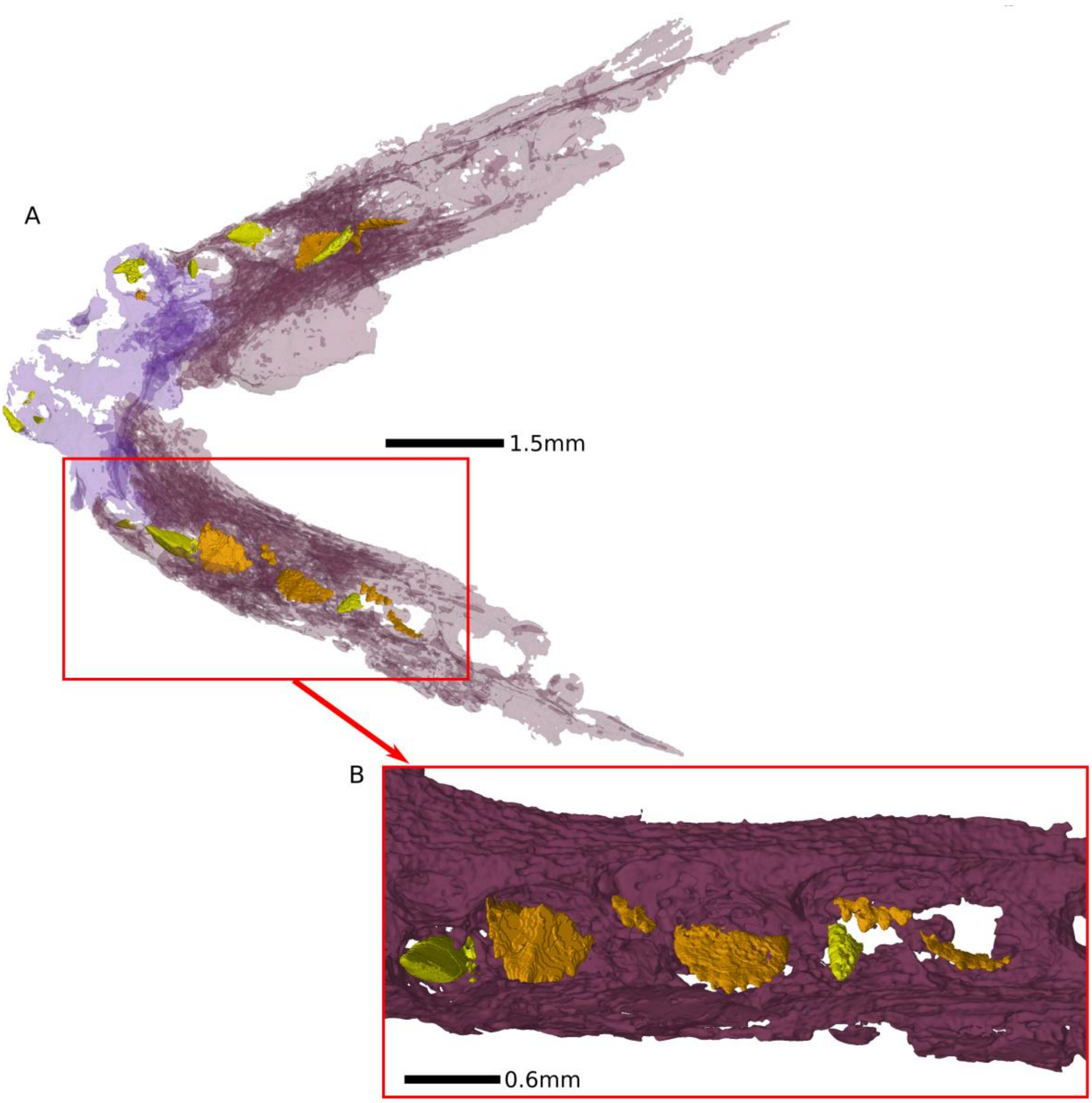

Figure S14. Positions of null generation teeth. A, maxilla of Embryo 2 in ventral view; B, close up of right maxilla of Embryo 2. Yellow teeth represent simple conical teeth (null generation teeth), orange teeth represent adult-morphology teeth.
